# A G-quadruplex-binding compound shows potent activity in human gemcitabine-resistant pancreatic cancer cells

**DOI:** 10.1038/s41598-020-68944-w

**Published:** 2020-07-22

**Authors:** Ahmed Abdullah Ahmed, Chiara Marchetti, Stephan A. Ohnmacht, Stephen Neidle

**Affiliations:** UCL School of Pharmacy, University College London, 29-39 Brunswick Square, London, WC1N 1AX UK

**Keywords:** Cancer, Chemical biology, Drug discovery

## Abstract

Gemcitabine is a drug of choice in the treatment of human pancreatic cancer. Chemo-resistance to this drug is common and has been attributed to a variety of distinct mechanisms, involving > 100 genes. A recently developed small-molecule G-quadruplex ligand, the trisubstituted naphthalene diimide compound CM03, has previously been shown to have equivalent potency to gemcitabine in the pancreatic cancer cell line MIA PaCa-2. We report here on cell lines of increased resistance to gemcitabine that have been generated from this line, with the most resistant having 1,000-fold reduced sensitivity to gemcitabine. These resistant lines retain nM sensitivity to CM03. The molecular basis for the retention of potency by this G-quadruplex ligand has been examined using whole transcriptome data analysis with RNA-seq. This has revealed that the pattern of pathways down regulated by CM03 in the parental MIA PaCa-2 cell line is largely unaffected in the gemcitabine-resistant line. The analysis has also shown that the expression patterns of numerous genes involved in gemcitabine sensitivity are down regulated in the resistant line upon CM03 treatment. These results are supportive of the concept that G-quadruplex small molecules such as CM03 have potential for clinical use in the treatment of gemcitabine-resistant human pancreatic cancer.

## Introduction

**Figure 1:**
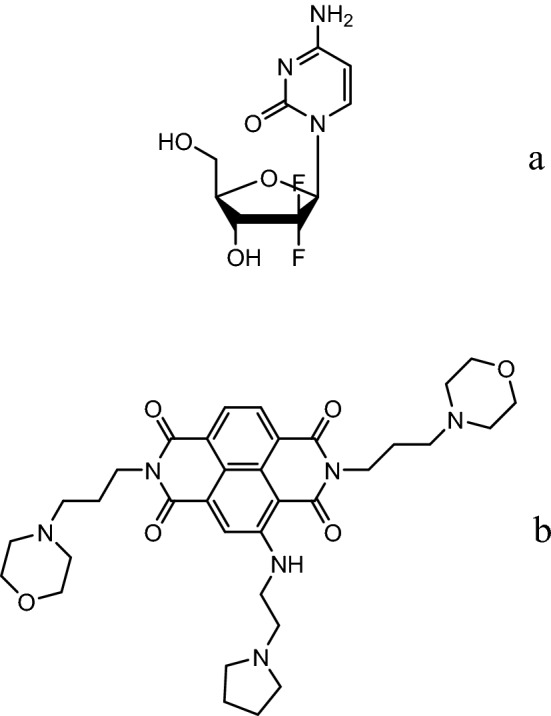
Structures of (**a**) gemcitabine, (**b**) CM03.

Pancreatic cancer is among the 12 most common cancers in the UK and the USA, with 9,921 new cases in the UK in 2015^1^ and 57,600 estimated new cases in the USA in 2020^2^. 458,918 new cases were reported world-wide in 2018^3^. Pancreatic ductal adenocarcinoma (PDAC: ca 85% of cases), is the most common form, and is also one of the most intractable of cancers to treatment. It has a bleak prognosis that has barely changed in over 20 years, with < 5% of patients surviving for five years^4–7^.The standard chemotherapy for PDAC has been the nucleoside analogue gemcitabine (Fig. 1a), which produces a modest improvement in mean survival of, typically, 2–3 months^8–10^. Initial responses are almost invariably followed by the rapid onset of chemo-resistance^11–14^. This has been attributed to, for example, changes in nucleoside transporter expression^15^, or in gemcitabine metabolising enzymes such as cytidine deaminase^16,17^. The complexity of the underlying mechanisms of gemcitabine resistance in PDAC is increasingly apparent and over 100 genes and multiple pathways may be involved^18–24^.

We have recently reported that several small-molecule naphthalene diimide derivatives^25,26^, notably the trisubstituted compound CM03^27^, are potent inhibitors of cancer cell growth, with CM03 (Fig. 1b) having a GI_50_ value of ca 11 nM in the PDAC cell lines PANC-1 and MIA PaCa-2. RNA-seq methodology has shown that CM03 targets a number of genes involved with PDAC initiation and progression in these cell lines, and also has significant anti-cancer activity in in vivo xenograft and genetic models for the disease. The mode of action of CM03 involves the stabilisation of genomic quadruplex DNA structures^28^ and as a consequence inhibits the expression of, in particular, those genes containing putative quadruplex-forming sequences (PQs) in their promoter regions^29–31^, and which have been identified as playing roles in PDAC^32–36^. By contrast, gemcitabine affects, in large part, a quite distinct set of genes, which are often not quadruplex-containing. It is also notable that CM03 shows in vivo anti-tumour activity^27^ in the KPC genetic mouse model, in which gemcitabine does not produce significant responses^37^. In the light of this evidence that CM03 and gemcitabine have distinct and orthogonal targets of action, we hypothesised that CM03 would show significant anti-proliferative activity in PDAC cell lines in which gemcitabine resistance has been generated. We report here the results of a study that addresses this concept. We have used the same approach as before^27^, examining data from RNA-seq analyses in order to be able to compare results with those from our previous study. We also discuss, in the light of the results presented here, the role that CM03 and related compounds could play in the clinic for the treatment of drug resistant PDAC.

## Results

### CM03 has sustained potent activity in gemcitabine-resistant pancreatic cancer cell lines

Gemcitabine-resistant (GemR) pancreatic cancer MIA PaCa-2 cell lines were generated for three different gemcitabine concentrations (0.25 µM, 1.0 µM and 3.0 µM), as described in the Methods section. The parental MIA PaCa-2 cells were designated as gemcitabine sensitive cells. The GemR cell lines were maintained at the three gemcitabine concentrations at which resistance was established. Prior to any experiment, gemcitabine was withdrawn from the culture medium for seven days. The resistant line at the highest gemcitabine concentration (3.0 µM), is the primary focus of the present study, and was designated as GemMIA-R3.

**Figure 2:**
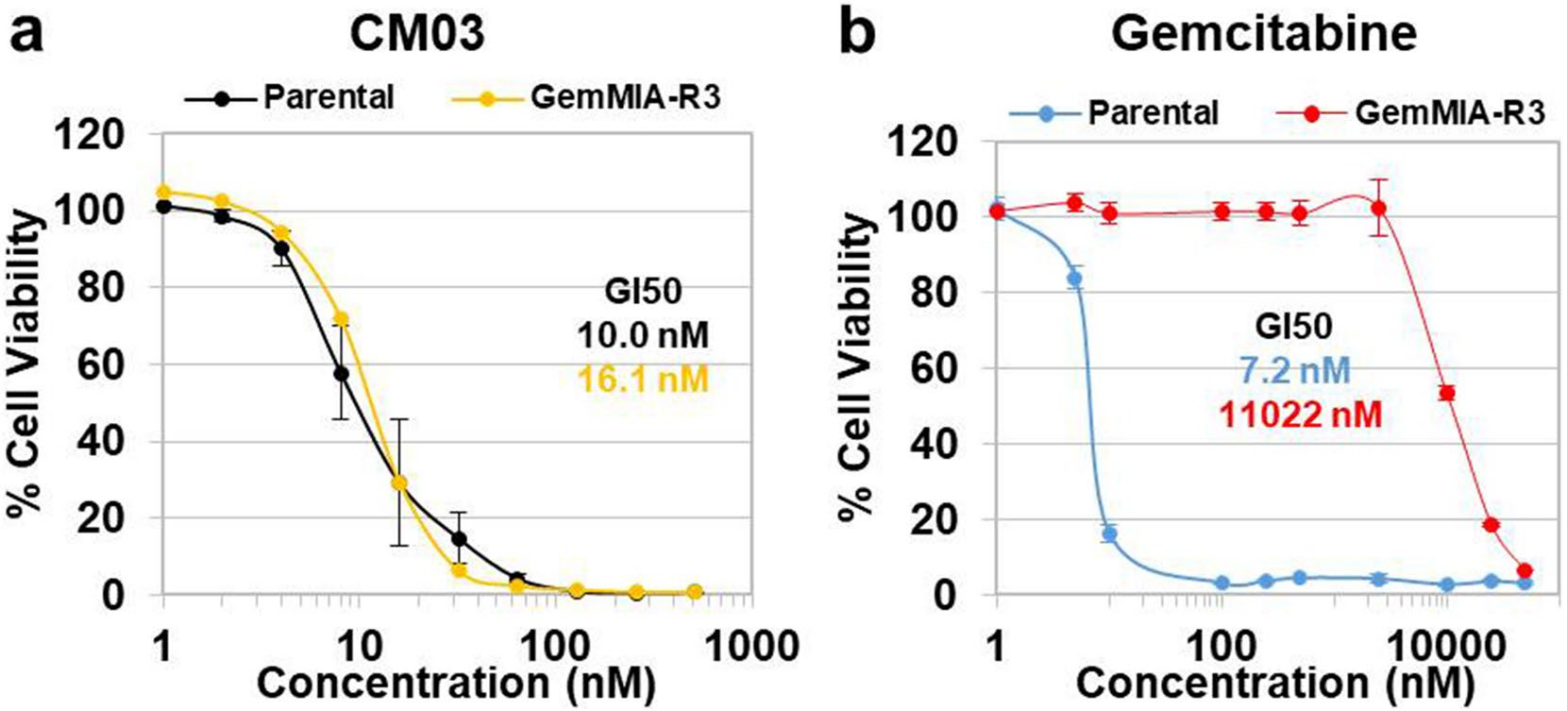
CM03 activity in parental and gemcitabine-resistant pancreatic cell lines. Dose–response curves of (**a**) CM03 and (**b**) gemcitabine in parental and gemcitabine resistant GemMIA-R3 cell lines. Cells of each cell line were seeded in triplicate in 96-well plate and incubated for 24 h. Then cells were treated with different concentrations of CM03 and gemcitabine for 96 h. After treatment, cells were fixed with 10% TCA and the cell viability was measured using the SRB assay. Data represent the mean ± SEM of at least three independent experiments.

To investigate the potency of CM03 on gemcitabine resistant GemMIA-R3 cells, growth inhibition assays were performed using a SRB (sulforhodamine B)-based method with a treatment period of 96 h, as described previously^38^ and as modified for use with quadruplex-binding small-molecule compounds^39^. The dose–response curves for CM03 are closely similar in both parental MIA PaCa-2 and GemMIA-R3 cells (Fig. 2a), in contrast to the results for gemcitabine with the two cell lines. In the latter instance the curve for GemMIA-R3 cells is shifted far to the right, indicating increased gemcitabine resistance (Fig. 2b). Gemcitabine dosing resulted in an GI_50_ value of 7.2 ± 0.7 nM in the parental cell line (Table 1), whereas the GI_50_ values in the resistant cell lines increased in line with the acquired gemcitabine resistance level continuing to rise (GemMIA-R0.25 < GemMIA-R 1< GemMIA-R3). The CM03 GI_50_ values remained comparable in all three GemMIA resistant cell lines to that in the parental cell line. This indicates that the mechanism of gemcitabine resistance does not influence the effects of CM03 on cell viability and by implication, on its mechanism of action. We have also examined the ability of the quadruplex ligand CX-5461^32,40^ to reduce growth in these two cell lines, in order to ascertain whether this structurally very dissimilar compound retains activity in both cell lines. We find that it has reduced cell-growth inhibitory activity compared to CM03 but similarly retains its activity in the GemMIA-R3 line (Table 1). This pattern of responses to gemcitabine, CM03 and CX-5461 has also been observed in the PANC-1 PDAC parental cell line and a gemcitabine-resistant line derived in the same multiple passage manner as the MIA PaCa-2 derived GemMIA-R3 one (Table S1).

**Table 1:** Cell growth inhibition data (GI_50_), in nM for parental and three gemcitabine-resistant MIA PaCa-2 cell lines.

Compound	Parental	GemMIA-R0.25	GemMIA-R1	GemMIA-R3
Gemcitabine	7.2 ± 0.7	2,404 ± 209	2,653 ± 121	11,022 ± 540
CM03	10.0 ± 2.8	9.7 ± 3.0	11.9 ± 3.5	11.2 ± 4.6
CX-5461	90.3 ± 30.7	–	–	88.7 ± 22.0

### The effect of gemcitabine resistance on CM03 mode of action

Since CM03 has equivalent potency in the parental MIA PaCa-2 and derived GemMIA-R3 cell lines (Table 1), it was hypothesised that the mode of action of CM03 as previously determined^27^ in the former would remain largely unaltered in GemMIA-R3 cells. In order to validate this hypothesis, a series of RNA-seq experiments were undertaken in order to profile transcriptome changes in the parental MIA PaCa-2 and GemMIA-R3 cell lines after 6 h and 24 h treatment with CM03.

In the absence of CM03 treatment, comparing GemMIA-R3 vs parental cells revealed a significant change in the gem-resistant cell line. There are ca 2,138 genes down-regulated (Log_2_FC < − 0.5, FDR < 0.1) and about 1,640 genes upregulated (Log_2_FC > 0.5, FDR < 0.1) in gem-resistant cells relative to the gem-sensitive parental line (Table 2). Such a broad difference in gene expression could change those genes targeted by CM03 and perhaps the mode of action as well.

**Table 2:** A summary list of differentially expressed genes (DEGs) in parental and gemcitabine-resistant GemMIA-R3 cells with and without CM03 treatment.

Cell lines	Compound/time	Down	Down strong	Up	Up strong
GemMIA-R3 vs Parental	–	2,138	984	1,640	774
Parental MIA PaCa-2	CM03_6h	2,203	617	2,219	861
	CM03_24h	2,272	770	2,495	1,054
GemMIA-R3	CM03_6h	939	248	647	212
	CM03_24h	2,694	1,187	2,892	1,383

Data from RNA-seq analysis on total RNA extracted from parental and GemMIA-R3 cell lines treated with 400 nM CM03 for 6 h and 24 h, in terms of numbers of DEGs. The DEGs are divided into four subsets: 1. Down Strong = Log_2_FC ≤ − 1 and FDR < 0.05; 2. Down = Log_2_FC < − 0.5 and FDR < 0.1; 3. Strong Up = Log_2_FC ≥ 1 and FDR < 0.05; 4. Up = Log_2_FC > 0.5 and FDR < 0.1.

**Figure 3:**
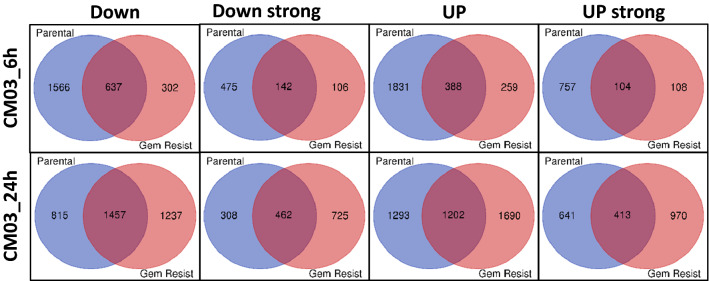
Venn diagrams comparing common DEGs between parental and gemcitabine resistant cell lines at 6 h and 24 h. The results of RNA-seq analysis on total RNA extracted from parental and GemMIA-R3 µM cell lines being treated with 400 nM CM03 for 6 h and 24 h, in terms of numbers of differentially expressed genes (DEGs). The DEGs are divided into four subsets: 1. Down Strong = Log_2_FC ≤ − 1 and FDR < 0.05; 2. Down = Log_2_FC < − 0.5 and FDR < 0.1; 3. Strong Up = Log_2_FC ≥ 1 and FDR < 0.05; 4. Up = Log_2_FC > 0.5 and FDR < 0.1.

The number of differentially expressed genes (DEGs) increased in both cell lines with duration of CM03 treatment although the numbers themselves varied (Table 2 and Fig. 3). The parental cell line was found to have a greater number of DEGs at 6 h than the GemMIA-R3 cell line while at 24 h this trend was reversed. This indicates a change in the response to more prolonged CM03 treatment, suggesting that it acts faster in parental gemcitabine-sensitive cells than in GemMIA-R3 ones.

Venn diagrams have been used to illustrate the number of genes with changes in expression that are in common between the two cell lines at 6 h and 24 h. Four different subsets of gene expression responses have been used (“down”, “down strong”, “up” and “up strong”). The definitions of these limits are given in the figure legend (Fig. 3). Overall, ca 30–65% of the altered genes are in common between the sensitive and resistant cell lines with CM03 treatment (Fig. 3). The expression profile of the GemMIA-R3 cells has undergone some change compared to the parental cells, which has resulted in an increase with respect to time of the down regulated (and also the up-regulated) genes common to the two cell lines. There are also increases in the numbers of genes that are not common to the two lines, based on their expression levels. For example, at 24 h 308 genes are highly down-regulated only in parental cells while 725 genes (among them some new CM03 targets) are highly down-regulated in GemMIA-R3 cells (Fig. 3).

**Figure 4:**
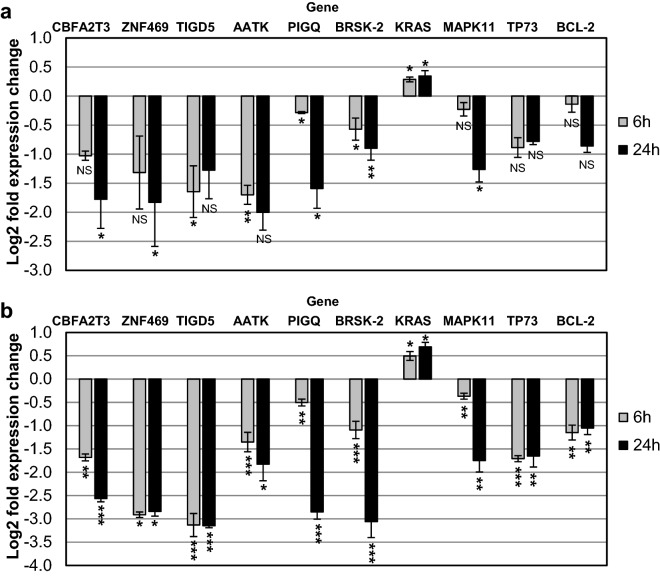
Variation in expression level of CM03 targeted genes in parental and gemcitabine resistant cell lines. (a) Parental and (b) GemMIA-R3 MIA PaCa-2 cell lines were treated with 400 nM CM03. RT-qPCR was performed for a subset of down-regulated genes, selected from RNA-seq experiments, shown above. The normalization of Ct values was done to the geometric mean of three housekeeping genes (*ACTB*, *GAPDH*, and *TUBB*), and the relative gene expression was determined using the Livak method, 2^−ΔΔCt^ . The log_2_FC for each gene is shown relative to control (vehicle PBS). Student’s t test was performed to determine the statistical significance of the observed changes, which are the mean of, in each case, at least three independent experiments, *P < 0.05, **P < 0.01, ***P < 0.005, NS = not significant.

To confirm the changes in CM03 targets between the parental and resistant cell lines, RT-qPCR was performed on a small set of previously-identified CM03 genes found to be down-regulated in the parental cell line, all of which contain PQs (Table 3)^27^. Cells were treated with 400 nM CM03 for 6 h and 24 h, i.e. using the same conditions as in the RNA-seq experiments. In general, the expression pattern for this panel of genes is strikingly similar for both parental and GemMIA-R3 cell lines, although the level of down-regulated expression is consistently greater in the GemMIA-R3 line, with most changes having **P < 0.01, using Student’s t-test. Notably, several genes highlighted in Fig. 4 such as *MAPK11*, *TP73* and *BCL-2* had expression changes which are not statistically significant or *P < 0.05 in parental cells yet were more significantly down-regulated in GemMIA-R3 cells. It is notable that the sole gene that is not down regulated in either cell line, *KRAS*, has the lowest number of PQs (Table 3).

**Table 3:** Number of PQs in the genes analysed in the RT-PCR study (Fig. 4).

Gene	No. of PQs
*CBFA2T3*	100
*ZNF469*	14
*TIGD5*	12
*AATK*	58
*PIGQ*	20
*BRSK2*	105
*KRAS*	6
*MAPK11*	24
*TP73*	96
*BCL-2*	21

PQ numbers were taken from the previous study on CM03^27^.

**Figure 5:**
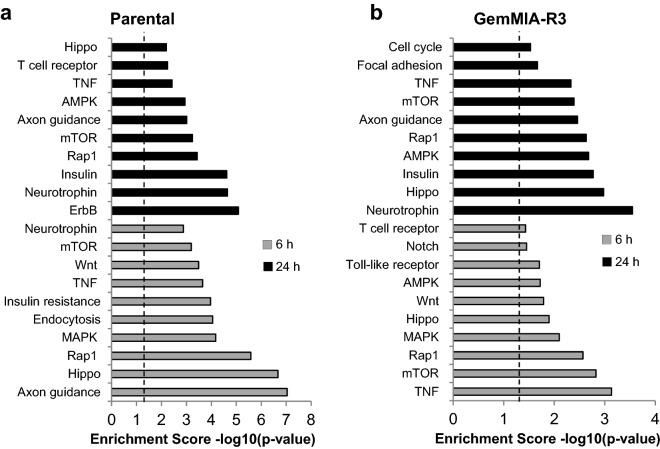
The top affected signalling pathways in parental and gemcitabine resistant MIA PaCa-2 cell lines after CM03 treatment. Parental and GemMIA-R3 cell lines were treated with 400 nM CM03 for 6 h and 24 h and the DEGs from RNA-seq analysis were used in signalling pathway enrichment analyses, using images from KEGG (by permission)^56–58^. Significantly enriched KEGG pathways (p-EASE ≤ 0.05) of down-regulated genes (Log_2_FC < − 0.5 and FDR < 0.1) for a. parental and b. gemcitabine-resistant (GemMIA-R3) cell lines after 6 h and 24 h treatment of 400 nM CM03. Above the dotted lines represent statistical significance of *P* value < 0.05, as calculated by Fisher’s exact test.

To check the effect of the change in CM03 targets on individual pathway responses, KEGG signalling pathway enrichment analyses were undertaken. The “down” subset of CM03 targets for both cell lines at 6 h and 24 h were used in these analyses. Figure 5 shows the most affected signalling pathways, which are closely similar in parental and GemMIA-R3 cells. These include the Hippo, mTOR, Rap1, MAPK and TNF pathways at 6 h of CM03 exposure as well as the Axon guidance, mTOR, Rap1, AMPK, neurotrophin, insulin and TNF pathways at 24 h exposure. Although the CM03 target pathways are not identical between the two cell lines, the overall pattern and therefore the key biological responses are closely similar, suggesting that the mechanism of CM03 cell growth inhibition in parental and resistant lines are also closely related.

**Figure 6:**
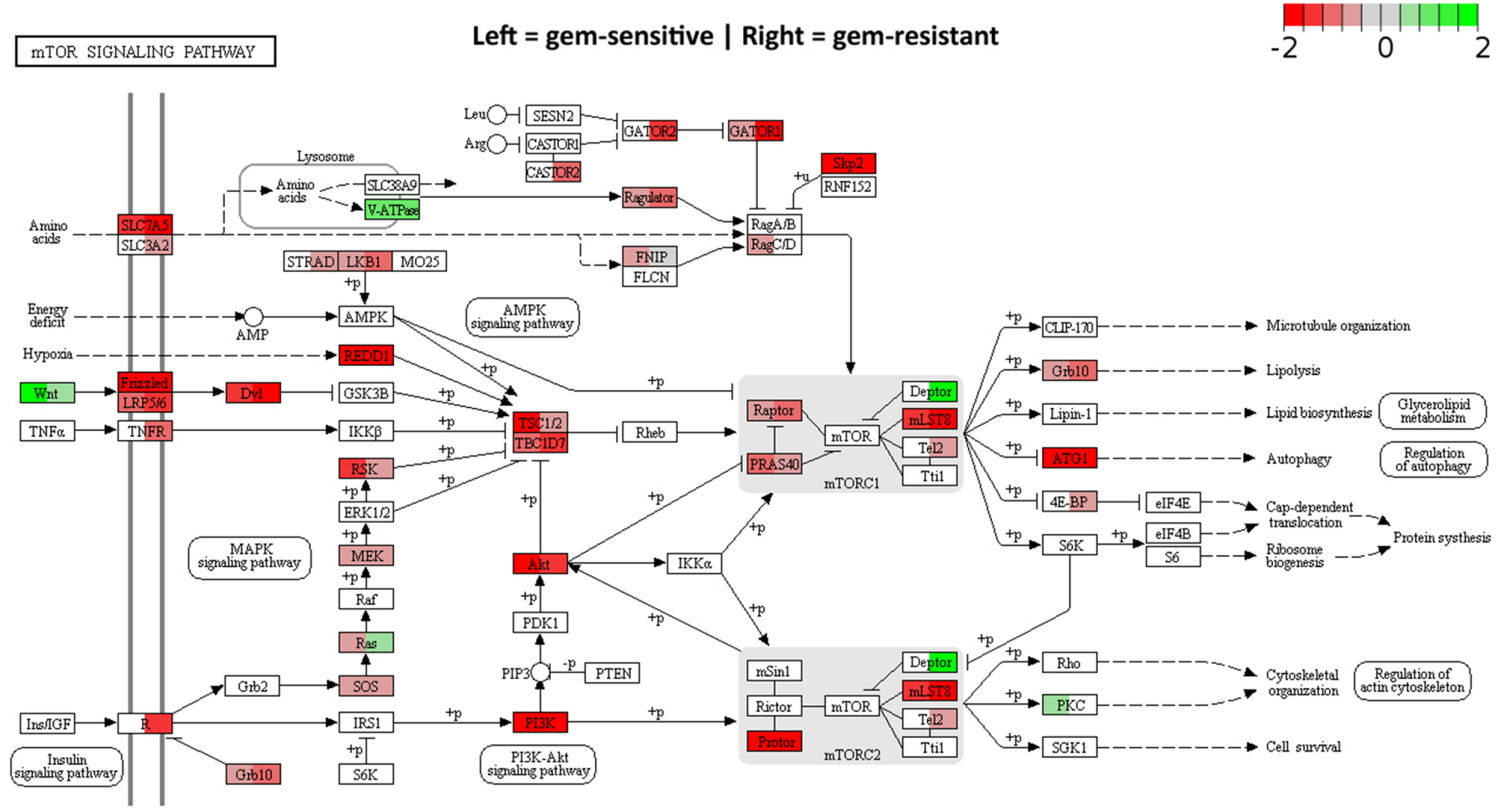
Significantly enriched KEGG pathways for a down-regulated gene set after 24 h CM03 treatment. KEGG pathway diagram illustrating significant DEGs (down = Log_2_FC < − 0.5 and FDR < 0.1, up = Log_2_FC > 0.5 and FDR < 0.1) in parental and GemMIA-R3 for the mTOR signalling pathway. Colours indicate log_2_ fold change of individual genes: red = downregulated < − 0.5, green = upregulated > 0.5 and grey between − 0.5 and 0.5. The left-hand side of each gene rectangle corresponds to the parental cell line and the right-hand side to the GemMIA line.

Figures 6, S1 and S2 show effects on five of these major pathways: mTOR, MAPK, Hippo, Axon guidance and Rap1. Multiple down-regulation effects are apparent, as has been previously found for CM03 in both MIA PaCa-2 and PANC-1 cells, but now extended to GemMIA-R3 cells. It is striking that the pattern of individual down-regulated genes in all five pathways is closely similar in the parental and resistant lines with a number of key pathway signalling genes having consistently down-regulated expression.

### CM03 down-regulate genes involved in gemcitabine sensitivity

Many genes have been implicated in gemcitabine resistance in MIA PaCa-2 cells, and in clinical gemcitabine resistance. The differential expression of a number of these genes has been examined here (Table 4). The data is mostly consistent with previous observations; genes that sensitize to gemcitabine were down-regulated in GemMIA-R3 cells (e.g. *dCK*), providing further evidence of the validity of the GemMIA-R3 line as authentically gemcitabine-resistant. Some genes that promote gemcitabine resistance were slightly up-regulated (for example, *RRM1&2* and *ABCC4*) and others were highly up-regulated (for example, *SHH* and *GLI1*). The effect of CM03 has been to down-regulate the expression of a number of these genes such as *SHH*, *GLI1*, *RRM2* and to a greater extent the *MAPK11*, *MAPK12* and *AKT1* genes. These genes are highly enriched in PQs and are consequently also important players in CM03 action in the parental line.

**Table 4:** A list showing several genes which have been reported as being implicated in gemcitabine resistance.

Gene ID	log_2_FC for GemMIA-R3 vs parental	log_2_FC for CM03 in parental cells	log_2_FC for CM03 in GemMIA-R3 cells	No. of PQs	Role in gemcitabine resistance	Refs.
*dCK*	− 2.99	− 0.37	− 0.30	2	The deoxycytidine kinase (dCK) enzyme metabolizes and activates gemcitabine	^13^
*CDA*	5.08	0.52	0.44	4	High level of cytidine deaminase (CDA) enzyme metabolizes gemcitabine leading to resistance	^16^
*RRM1*	0.70	− 0.38	− 0.09	7	The overexpression of ribonucleoside-diphosphate reductase M1 and M2 (RRM1/2) genes reduce gemcitabine metabolism, leading to resistance	^18^
*RRM2*	0.34	− 0.48	− 0.50	3		
*SHH*	3.50	0.86	− 0.16	17	Deregulated hedgehog pathway is associated with gem-resistance where SHH ligand is overexpressed	^14^
*GLI1*	3.60	− 0.64	− 0.57	15	GLI transcription factors promote gem-resistance via *SOX2* overexpression	^23^
*ABCC4*	0.77	0.02	− 0.05	28	Overexpressed multidrug resistance gene, increases drug efflex	^15^
***MAPK11***	**0.00**	**− 2.56**	**− 3.03**	**18**	**MAPK signalling pathway**	^19^
***MAPK12***	**0.05**	**− 1.23**	**− 1.83**	**18**		^19^
***AKT1***	**0.10**	**− 0.98**	**− 1.55**	**44**	**AKT signalling pathway**	^14^
*FOXM1*	− 0.16	− 0.13	− 0.52	2	Oncogenic transcription factor which is associated with gem-resistance	^14^
*ST6GAL1*	2.84	− 0.11	− 1.16	21	Promotes gem-resistance by abolishing gem-mediated DNA damage	^21^
*FASN*	0.31	− 0.54	− 1.17	40	Increase in fatty acid synthase (*FASN*) expression correlates with increased gem-resistance	^20^

The effects of CM03 on their expression in the parental and resistant GemMIA-R3 cells (following 24 h exposure to CM03) are shown. There are many changes in expression for genes associated with gemcitabine resistance. There is also a trend for the genes previously identified with CM03-induced down-regulation in the parental cells (highlighted in bold), to be more down-regulated in the GemMIA-R3 line. The number of putative quadruplexes (PQs) in each gene were taken from the earlier study^27^, computed as reported previously^52,53^ and using the occurrence of the canonical G4 motif (G_≥3_N_1−7_G_≥3_N_1−7_G_≥3_N_1−7_G_≥3_).

The gene for the rate limiting enzyme deoxycytidine kinase (dCK) in gemcitabine metabolism, which metabolizes and activates gemcitabine^10^ is highly down-regulated (log_2_FC = − 2.99) in GemMIA-R3 cells, in accordance with the high level of resistance in this line. In addition, expression of the cytidine deaminase gene *CDA* (coding for the enzyme that inactivates gemcitabine upon transport into cells^16^, is highly upregulated (log_2_FC = 5.08), further increases chemo-resistance to gemcitabine (Table 4). CM03 has only a minor effect on the expression of these two genes, both in the parental and the GemMIA-R3 line: this is in accord with the small number of predicted PQs in both genes. The over-expression of the *RRM1* and *RRM2* ribonucleoside-diphosphate reductase sub-unit genes is another established contributor^18^ to gemcitabine resistance: their up-regulation was also observed in GemMIA-R3 cells. CM03 treatment of GemMIA-R3 cells down-regulates *RRM2* by ca 30%, which may lower gemcitabine resistance.

The hedgehog (Hh) signalling pathway has also been found to play a role in promoting gemcitabine resistance^14^, as well as GLI transcription factors, by upregulating SOX2 signalling^23^. Two key genes in the Hh pathway, sonic hedgehog ligand (SHH) and transcription factor GLI1 are highly up-regulated in GemMIA-R3 cells, with log_2_FC values of 2.5 and 3.6, respectively. These two genes, which have high PQ representation, are slightly downregulated by CM03 but not statistically significantly. The *MAPK11*, *MAPK12* and *AKT1* genes are not upregulated in the GemMIA-R3 cell line, whereas their expression is highly down-regulated upon CM03 treatment (Table 4).

Analogous behaviour has been found for the *ST6GAL1* gene (Table 4), which encodes for β-galactoside α-2,6-sialyltransferase-1. Knockdown of this enzyme sensitizes cells to gemcitabine by increasing cell death and DNA damage^21,40^ and it notable that CM03 treatment results in an increase in the number of DNA damage foci^27^. The *ST6GAL1* gene is highly over-expressed (log_2_FC = 2.84) in the GemMIA-R3 cell line, presumably contributing to its high level of gemcitabine resistance. CM03 treatment results in significant down-regulation of *ST6GAL1* (log_2_FC = − 1.16) in GemMIA-R3 cells compare to its small effect in parental cells (log_2_FC = − 0.11). This large difference may be because that the *ST6GAL1* gene is more accessible in gem-resistant cells than in sensitive cells. CM03-mediated downregulation of ST6GAL1 should reduce or reverse the effect of overexpressed ST6GAL1 on increasing chemo-resistance.

The Forkhead box protein M1 (FOXM1) is an oncogenic transcription factor and its elevated expression is associated with gemcitabine resistance in patients with pancreatic cancer^24^. Expression of the *FOXM1* gene did not significantly alter in the GemMIA-R3 cell line compared to the parental line, but CM03 treatment results in some downregulation (log_2_FC = − 0.52).

Elevated expression of fatty acid synthase (FASN) has been correlated with reduced response to gemcitabine and inhibition of FASN results in synergistic effects on gemcitabine treatment^20^. In accord with this, *FASN* gene expression level is slightly increased in the GemMIA-R3 cell line. The *FASN* gene contains 40 PQs (Table 4) which makes it a potential target for CM03: *FASN* expression was decreased upon CM03 treatment in both parental and GemMIA-R3 cell lines (log_2_FC = − 0.54 and − 1.17, respectively).

## Discussion

A cell line (GemMIA-R3) has been derived from the parental MIA PaCa-2 PDAC cell line, with ca 1,000- fold resistance to the clinically used drug gemcitabine, using a repeated passage approach. Its validity as a resistant line is supported by the changes in expression for several genes associated with gemcitabine resistance, notably *DCK*, *CDA*, *GLI1* and *ST6GAL1*.

The G-quadruplex small-molecule CM03 compound has ca 10 nM potency (GI_50_ for cell growth inhibition) in parental MIA PaCa-2 cells. Its potency is fully retained in the GemMIA-R3 line, as well as in an equivalent gemcitabine resistant line derived from the PANC-1 PDAC line, indicating that potency is not restricted to a single PDAC resistant line, and may be a more general phenomenon. The structurally unrelated G-quadruplex compound CX-5461 is also equipotent in parental and resistant lines, suggesting that the mechanism of action of this G-quadruplex compound also involves distinct genes and pathways to gemcitabine and its resistance.

RNA-seq whole transcriptome analysis has previously been used to establish that compound CM03 down-regulates the expression of multiple cancer-related genes and pathways in two PDAC cell lines, MIA PaCa-2 and PANC-1, and that these targets are mostly enriched with quadruplex sequences. The inference is that multiple quadruplexes are the targets of CM03, which may confer therapeutic advantage in a complex human cancer such as PDAC^32,41,42^, where mutational complexity and genomic instability increase with disease progression. It is shown here that the pattern of gene and pathway down-regulation following CM03 treatment in parental PDAC cell lines is in large part maintained in the resistant line GemMIA-R3, hence the potency of CM03 in this line, and by inference in the resistant PANC-1 line. Further studies with appropriate in vivo models will be needed in order to validate CM03 as a potential drug for the treatment of chemoresistant PDAC in humans. The present cell-based study does also suggest that several genes such as *dCK*, *CDA* and *ST6GAL1* may be useful prognostic markers of this disease^43^.

Several other quadruplex ligands have been previously reported as having activity in chemo-resistant cell lines^44–47^, for example substituted naphthalene diimide-based in patient-derived gastrointestinal cancer cells^44^ and in BRAF-mutant melanoma cells^45^. We speculate that these and perhaps other quadruplex ligands^46,47^ show such activity as a result of their ability to down-regulate multiple genes, which are at least in part distinct from those conferring resistance. The present results do show that the expression of some resistance genes (such as *ST6GAL1*), as well as some that are in common with those involved in CM03-induced down-regulation of proliferation and resistance (such as *GLI1*), is down-regulated by CM03 treatment. This suggests that CM03 treatment of gemcitabine-resistant cells could result in a reduction in gemcitabine resistance. This concept has yet to be evaluated.

## Methods

### Chemicals

Gemcitabine as the hydrochloride salt was purchased from Sigma-Aldrich (cat no. G6423). CX-5461 was purchased from Adooq Bioscience (cat no. A11065). Compound CM03 was synthesised in-house^27^ and was used as the > 95% pure hydrochloride/formate salt. This has a pH of 6.95 in H_2_O. Gemcitabine, CM03 salts and CX-5461 were dissolved in PBS and stocks of 1 mM were prepared and kept frozen and away from light prior to use. Drugs were filtered through 0.22 µm pore-size filter units before addition to appropriate cell culture media.

### Cell culture and growth inhibition

MIA PaCa-2 and PANC-1 cell lines were purchased from ATCC (cat no. CRL-1420 and CRL-1469) and maintained in Dulbecco's Modified Eagle's Medium (DMEM) with 4,500 mg/L glucose and 2 mM L-glutamine (Sigma-Aldrich, cat no. D6429) supplemented with 10% foetal bovine serum (ThermoFisher, cat no. 10270106), 0.1 mg/ml streptomycin and 100 U/ml penicillin (Sigma-Aldrich, cat no. P4333) and only for MIA PaCa-2 cells: 2.5% horse serum (ThermoFisher, cat no. 16050130). Cells were maintained at 37 °C and 5% CO_2_ in humidified incubators and routinely passaged.

Cellular growth inhibition was measured using the sulforhodamine B (SRB) assay in 96 well plates as described previously^38,39^. 50% growth inhibition (GI_50_ values) were determined by taking the mean absorbance at 540 nm for each drug concentration expressed as a percentage of the absorbance of untreated control wells. All experiments were performed in triplicate and the mean ± SD values were determined from at least three independent experiments.

### Generation of gemcitabine-resistant cell lines

Gemcitabine-resistant cell lines were generated from the parental MIA PaCa-2 pancreatic cancer line by incrementally increasing the gemcitabine concentration in the culture medium over extended periods of time. A cycle of concentration increase and selection was repeated until reaching a particular target concentration (i.e. 0.25 µM, 1.0 µM or 3.0 µM). Each increment of gemcitabine concentration took around a week for cells to gain resistance to and resume cell proliferation. The whole process took several months to achieve the most resistant cell line for the highest gemcitabine concentration (3 µM). At each point, the cells were amplified, cryopreserved and maintained continuously in medium containing gemcitabine at the concentration of interest. The gemcitabine selection pressure was withdrawn for 7 days before a particular gemcitabine-resistant cell line was used in any experiment, in order to avoid any interference from gemcitabine.

### RNA-seq analysis

GemMIA-R3 cells were seeded in 100 mm plates (6 h = 2.5 × 10^6^ and 24 h = 1.0 × 10^6^ cells/well) and incubated overnight. Then, cells were treated with 0.40 µM CM03 or vehicle (PBS) for 6 h and 24 h. This is the lowest concentration of CM03 that can achieve minimal cell death (10%) and was chosen for these short-term exposures, in order to investigate its direct gene targets and not collateral gene expression changes. It was the same as previously used^27^, maintaining consistency with the previous study in which it had been shown to cause substantial gene expression changes even after 6 h treatment. Parental cells were also included without CM03 treatment for GemMIA-R3 vs parental gene expression comparison. Total RNA was extracted from 2–4 × 10^6^ cells using the RNeasy mini kit (Qiagen, cat no. 74104) and on-column DNase1 digestion (Qiagen, cat no. 79254) as per the manufacturer’s instructions. RNA quality (RIN > 7.0) was checked with an Agilent 2,100 Bioanalyser RNA 6,000 Nano Chip and RNA concentration was quantified using a Qubit fluorometer (ThermoFisher) and Qubit RNA HS Assay Kit (ThermoFisher, cat no. Q32852). RNA-seq libraries were then generated using the NEBNext mRNA Ultra II with IDT xGen UMI adapters kit for Illumina as per the manufacturer’s instructions and sequenced using an Illumina NextSeq 500 instrument (undertaken at the UCL Genomics Facility).

### RNA-seq data processing

The raw and processed sequencing data has been deposited in the GEO public functional genomics data repository with GEO accession no GSE148200 (https://www.ncbi.nlm.nih.gov/geo/). RNA-seq data (GEO accession GSE105083) from our previous study with CM03 and the parental cell line MIA PaCa-2 was used for comparison purposes during the analyses^27^. Illumina run data were demultiplexed and converted to fastq files using Illumina’s bcl2fastq Conversion Software (v2.19). Then, fastq files were pre-processed to remove adapter contamination and poor quality sequences using the program Trimmomatic (v0.36)^48^ before being mapped to a recent human genome build (UCSC hg38: https://genome.ucsc.edu/) using the RNA-seq alignment tool STAR (v2.5b: https://github.com/alexdobin/STAR). Duplication levels were estimated using JE-Suite^49^, a Unique Molecule Identifier program to filter out duplicates and reads that are the result of PCR amplification were marked. Next, reads per transcript were counted using the program FeatureCounts^50^ (v1.4.6p5) before normalisation, modelling and differential expression analysis using the SARTools (v1.3.2) package^51^.

For the numbers of putative G4 sequences in an individual gene (PQs in Table 4), the occurrence of the canonical G4 motif (G_≥3_N_1−7_G_≥3_N_1−7_G_≥3_N_1−7_G_≥3_), was used as previously reported^27^, in gene promoters (defined for this purpose as being up to 2 kilobases upstream of the transcription start site (TSS) and 100 bases downstream) and in exons and introns. These numbers of putative quadruplexes (PQs) were taken from the earlier study^27^, computed as reported previously^52,53^.

Differentially expressed genes (DEGs) were split into different subsets according to their log_2_ fold changes (Log_2_FC) and false discovery rate (FDR) for drug treatment versus untreated, with the following assignment: Down Strong = genes with Log_2_FC ≤ − 1 and FDR < 0.05; Down = genes with Log_2_FC < − 0.5 and FDR < 0.1; Strong Up = genes with Log_2_FC ≥ 1 and FDR < 0.05; Up = genes with Log_2_FC > 0.5 and FDR < 0.1. The signalling pathway enrichment analysis was done using the DAVID functional annotation tool (https://david.ncifcrf.gov/)^54^ on the Up and Down gene lists. The Pathview maps tool (https://pathview.uncc.edu/)^55^ was used to visualise top affected signalling pathways containing coloured DEGs from Up and Down gene lists to indicate their level of expression.

### RT-qPCR study

Parental MIA PaCa-2 and GemMIA-R3 cell lines were seeded and treated exactly same as in RNA-seq experiment. After extracting total RNA, the RNA concentrations were measured with a NanoDrop 2000/2000c spectrophotometer (ThermoFisher). The cDNA libraries were then prepared from a determined amount of RNA using a SuperScript III First-Strand Synthesis System (ThermoFisher, cat no. 18080051) as per the manufacturer’s instructions. RT-qPCR was performed using Power SYBR Green Master Mix (ThermoFisher, cat no. 4368706) with a 50 ng template and 150 nM primers in an AriaMx Realtime PCR System (Agilent). Primers were purchased from Sigma (KiCqStart SYBR Green Primers) and Eurofins Genomics. At least three independent experiments were carried out in triplicate. The data was used to determine the Ct values, which were normalized to the geometric mean of three housekeeping genes *ACTB*, *GAPDH*, and *TUBB*, and the fold change was determined using 2^−ΔΔCt^.

## Supplementary information

Supplementary information
