# Supplementary material for: A G-quadruplex-binding compound shows potent activity in human gemcitabine-resistant pancreatic cancer cells
Source: Sci Rep. 2020 Jul 22;10:12192. doi: 10.1038/s41598-020-68944-w (PMC7376204; doi:10.1038/s41598-020-68944-w)
Supplement: Supplementary file 1 — Supplementary information [file 41598_2020_68944_MOESM1_ESM.docx]

Supplementary data

A G-quadruplex-binding compound shows potent activity in human gemcitabine-resistant pancreatic cancer cells

Ahmed Abdullah Ahmed^1^, Chiara Marchetti^1^, Stephan Ohnmacht^1^ and Stephen Neidle^1^*

*^1^ UCL School of Pharmacy, University College London, 29-39 Brunswick Square, London WC1N 1AX, UK*

Corresponding author: [s.neidle@ucl.ac.uk](mailto:s.neidle@ucl.ac.uk)

**Supplementary Table S1**

Cell growth inhibitory data for parental PANC-1 and gemcitabine-resistant PANC-1 cell lines, obtained by multiple passages with gemcitabine at increasing concentration until reaching 3 µM exposure. GI_50_ values are in nM.

| **Compound** | **PANC-1 Parental** | **PANC-1 GemResist 3 µM** |
| --- | --- | --- |
| **Gemcitabine** | 23.3 ± 8.4 | 28750.9 ± 6121.3 |
| **CM03** | 10.4 ± 1.2 | 15.5 ± 1.8 |
| **CX-5461** | 32.9 ± 7.6 | 58.8 ± 13.8 |

**Supplementary Figures:**

**Figures S1 and S2. Significantly enriched KEGG pathways for down-regulated gene sets after 24 h CM03 treatment.**

KEGG pathway diagrams illustrating significant DEGs (down = Log_2_FC < -0.5 and FDR <0.1, up = Log_2_FC > 0.5 and FDR <0.1).

Colours indicate log_2_ fold change of genes: red = down-regulated < -0.5, green = upregulated > 0.5 and grey between -0.5 and 0.5.

**Figure S1.** (a, c) Gem-sensitive and (b, d) gem-resistant cell lines for (a, b) MAPK and (c, d) Hippo signalling pathways.


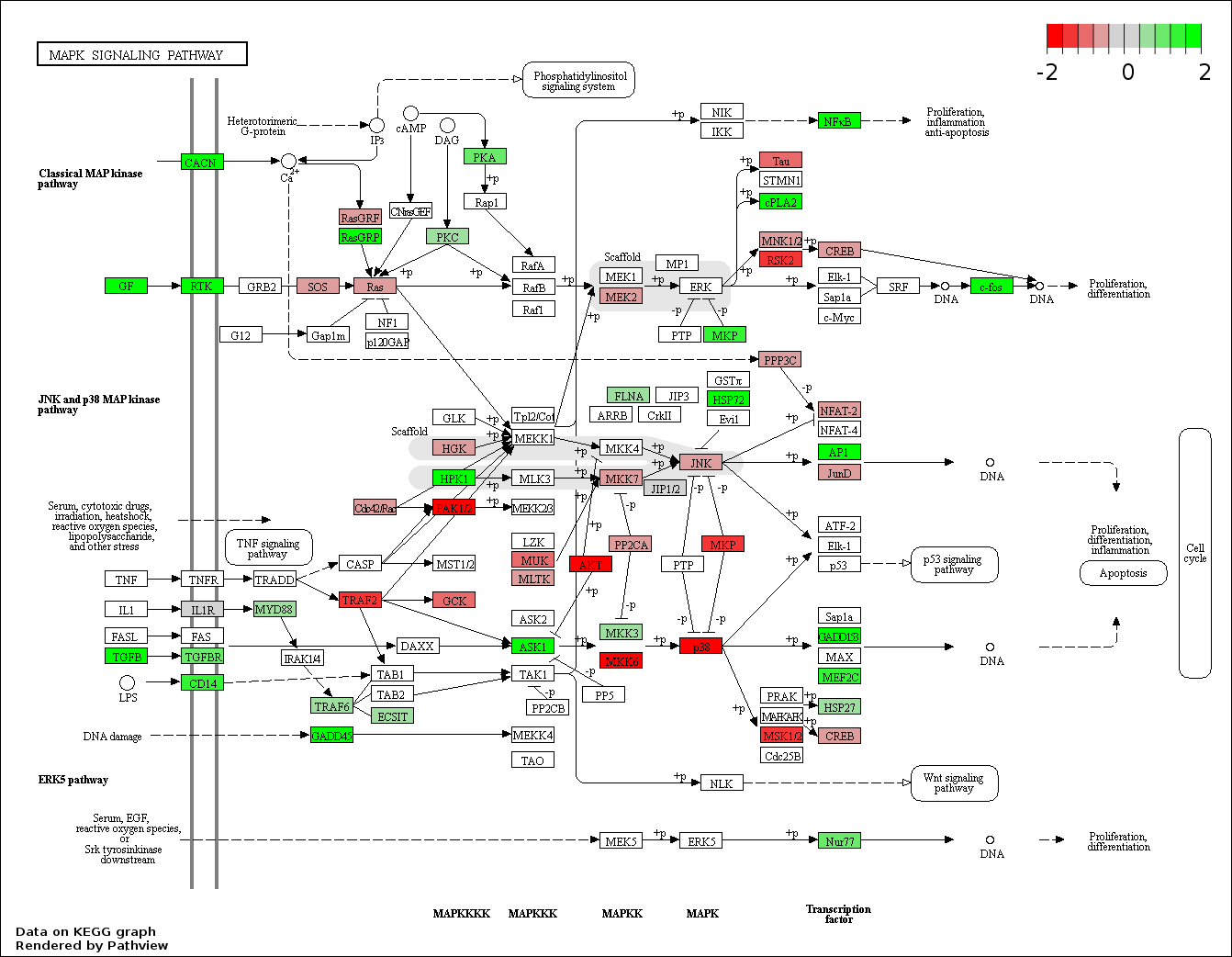
**a**


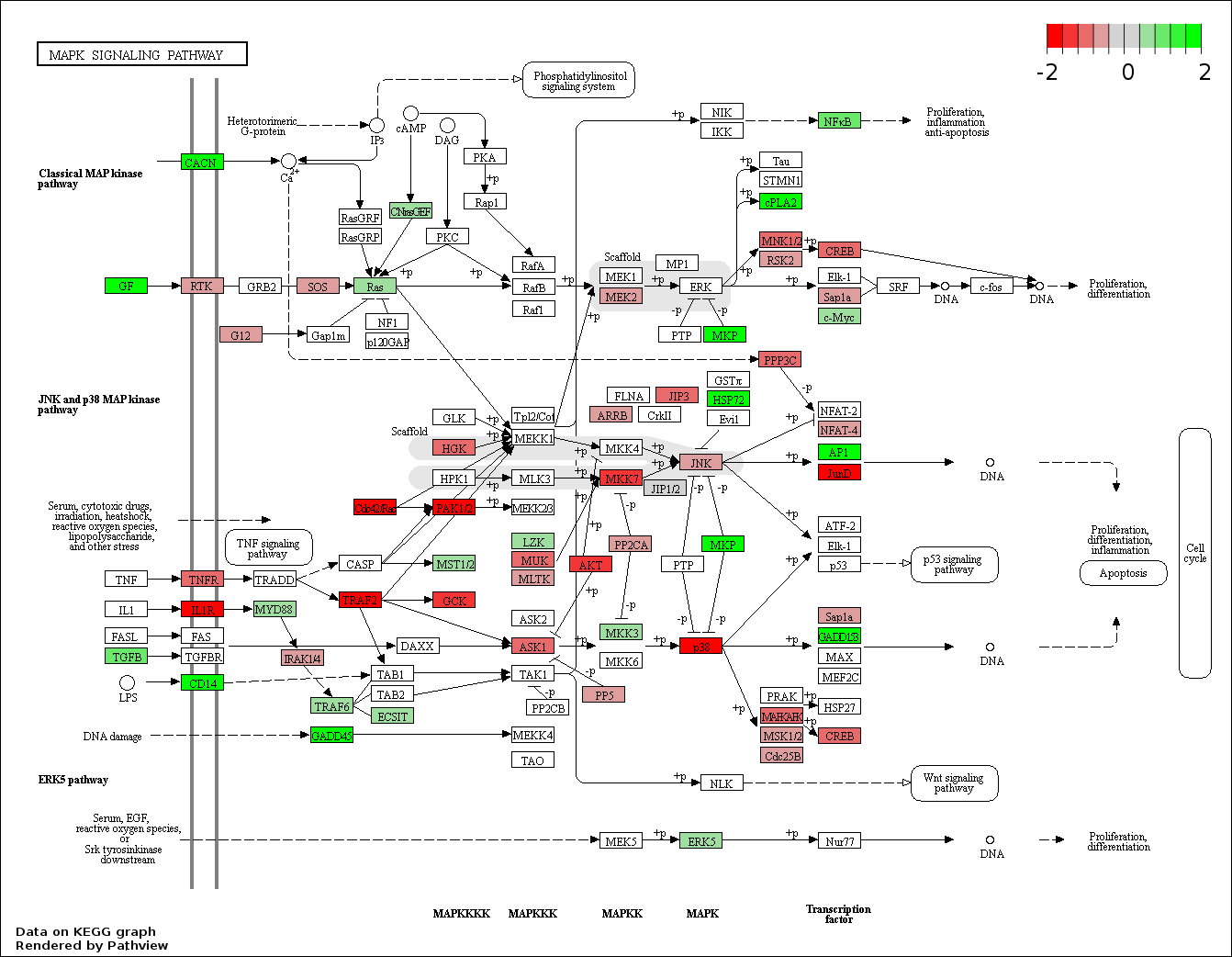
**b**


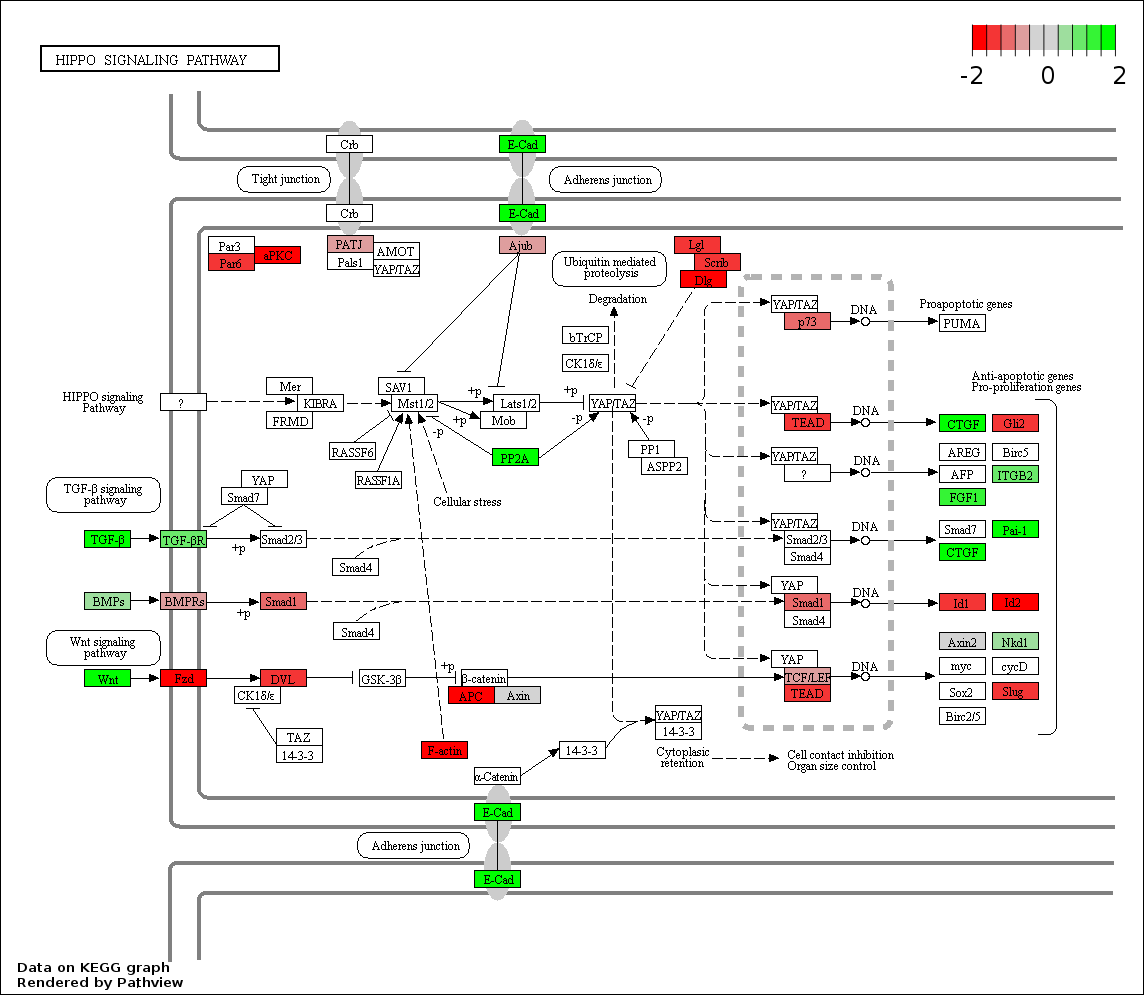
**c**

**d**


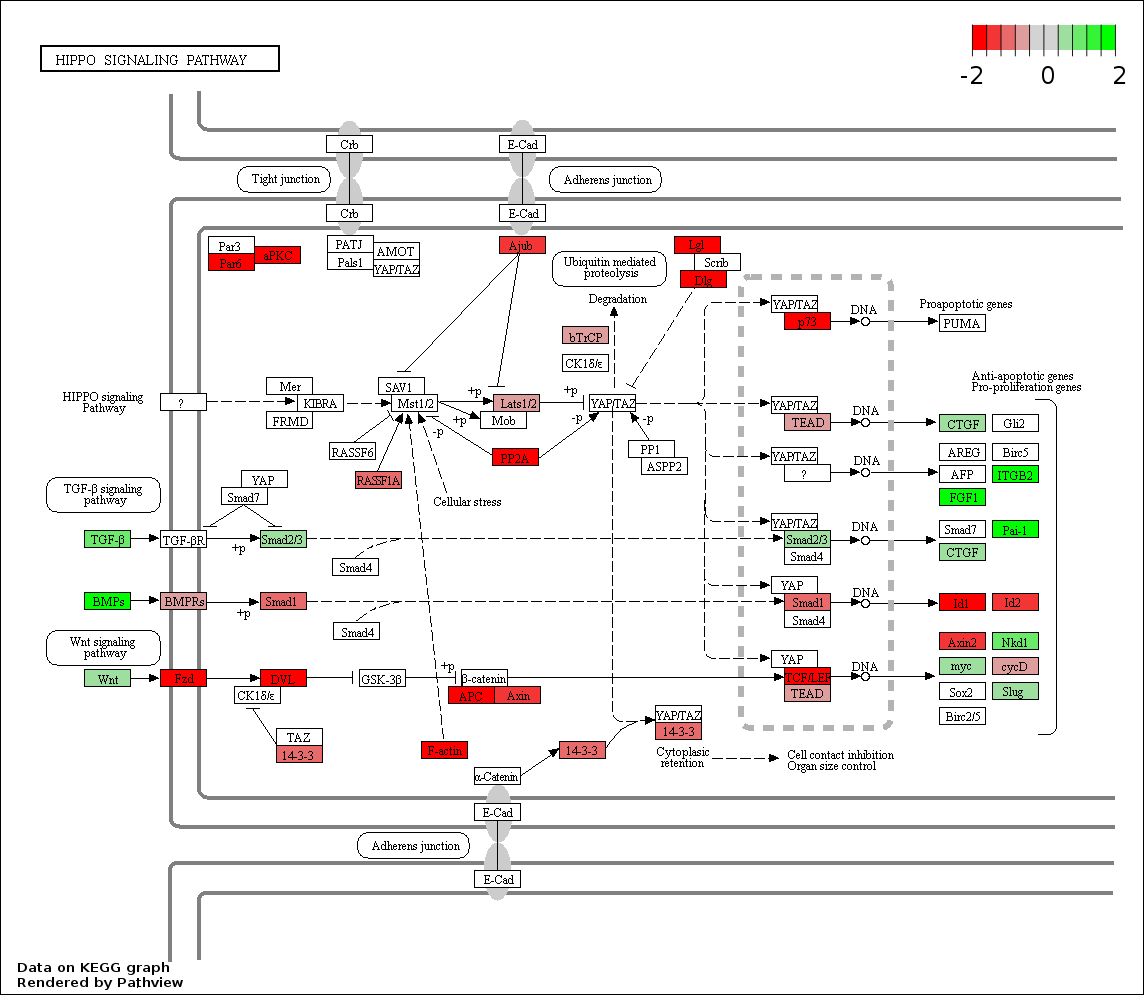


Figure S2. (a, c) Gem-sensitive and (b, d) gem-resistant cell lines for (a, b) Axon guidance and (c, d) Rap1 signalling pathways.


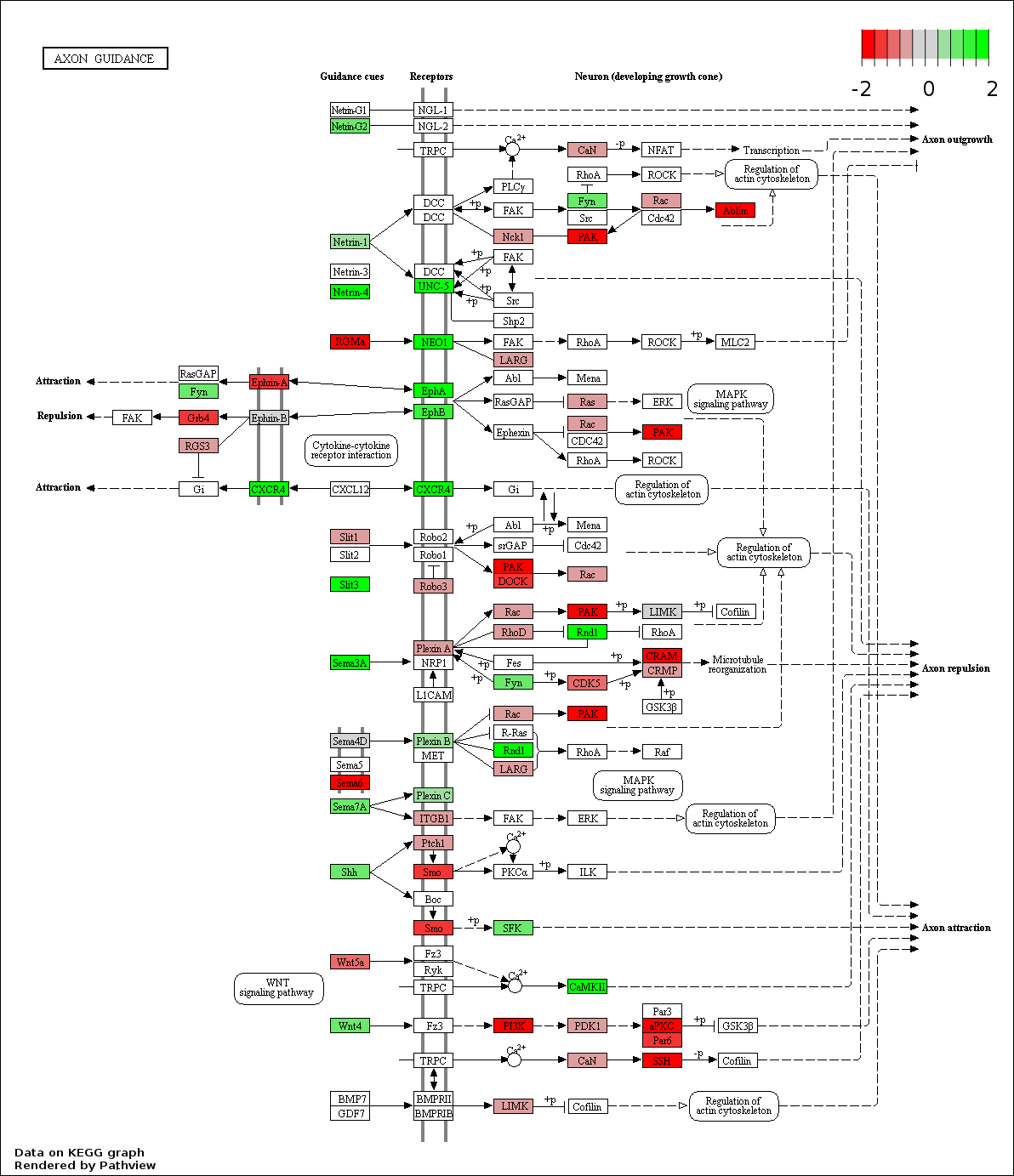
**a**


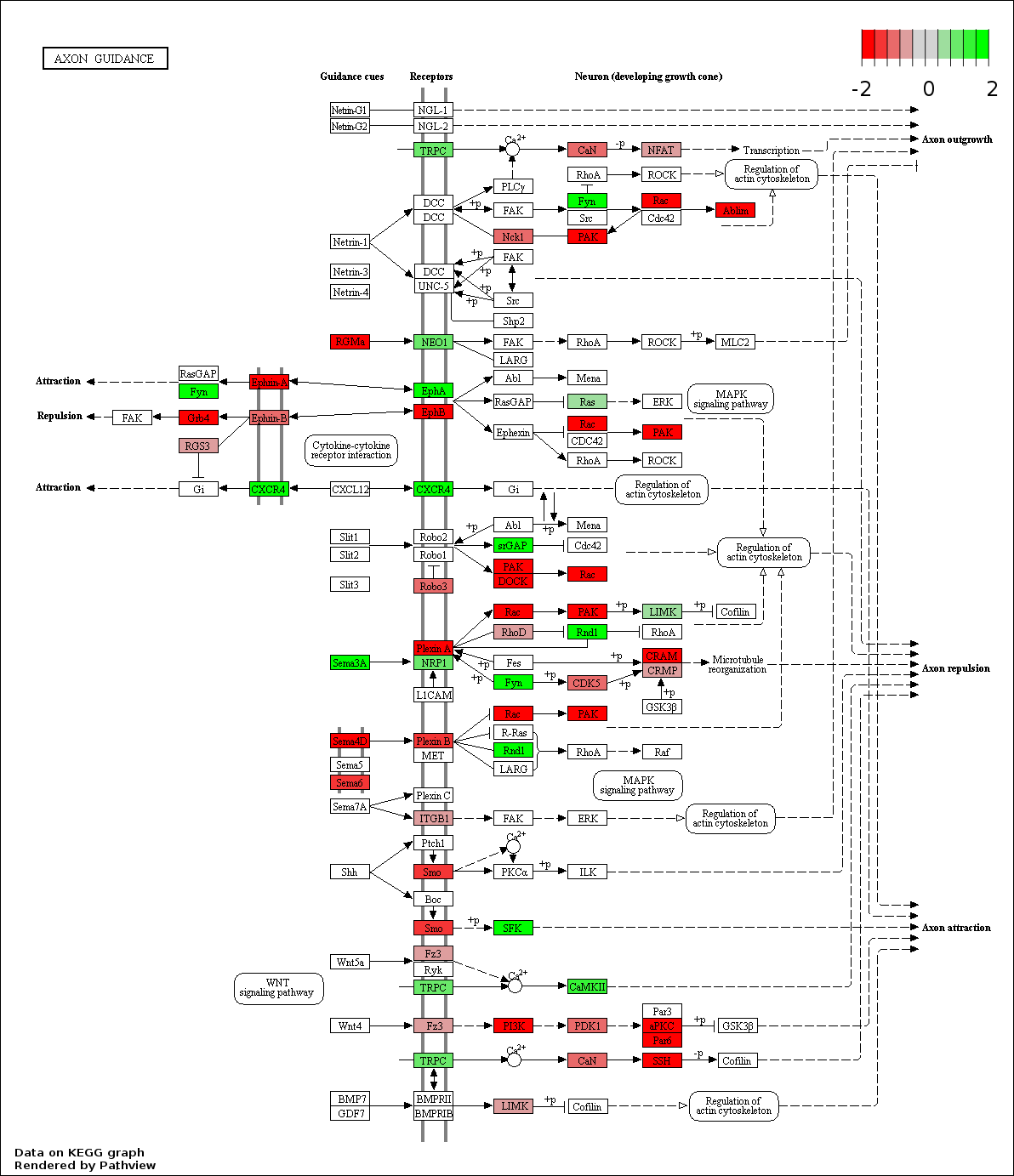
**b**

**c**


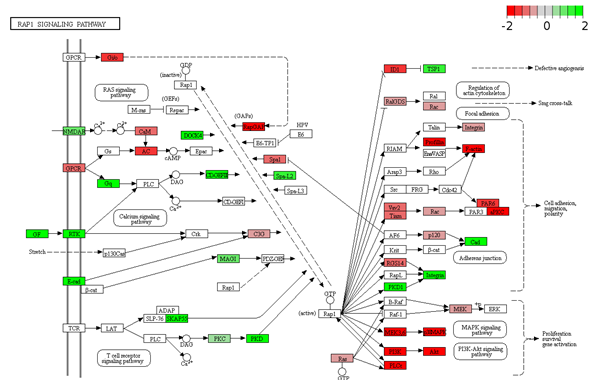


**d**


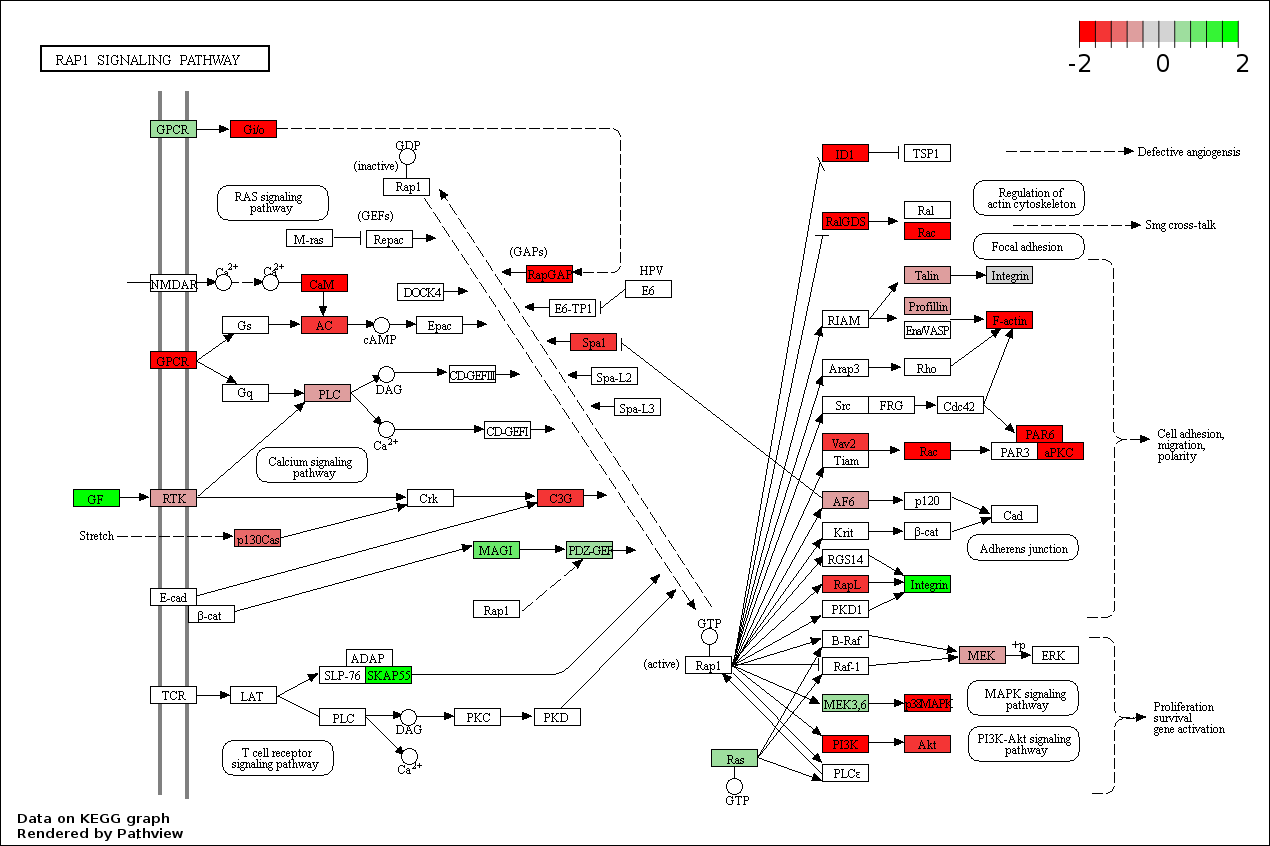


d
